# Self-supervised pretraining improves the performance of classification of task functional magnetic resonance imaging

**DOI:** 10.3389/fnins.2023.1199312

**Published:** 2023-06-26

**Authors:** Chenwei Shi, Yanming Wang, Yueyang Wu, Shishuo Chen, Rongjie Hu, Min Zhang, Bensheng Qiu, Xiaoxiao Wang

**Affiliations:** ^1^Center for Biomedical Imaging, University of Science and Technology of China, Hefei, Anhui, China; ^2^Institute of Artificial Intelligence, Hefei Comprehensive National Science Center, Hefei, China

**Keywords:** fMRI, deep learning, self-supervised, interpretability, brain networks

## Abstract

**Introduction:**

Decoding brain activities is one of the most popular topics in neuroscience in recent years. And deep learning has shown high performance in fMRI data classification and regression, but its requirement for large amounts of data conflicts with the high cost of acquiring fMRI data.

**Methods:**

In this study, we propose an end-to-end temporal contrastive self-supervised learning algorithm, which learns internal spatiotemporal patterns within fMRI and allows the model to transfer learning to datasets of small size. For a given fMRI signal, we segmented it into three sections: the beginning, middle, and end. We then utilized contrastive learning by taking the end-middle (i.e., neighboring) pair as the positive pair, and the beginning-end (i.e., distant) pair as the negative pair.

**Results:**

We pretrained the model on 5 out of 7 tasks from the Human Connectome Project (HCP) and applied it in a downstream classification of the remaining two tasks. The pretrained model converged on data from 12 subjects, while a randomly initialized model required 100 subjects. We then transferred the pretrained model to a dataset containing unpreprocessed whole-brain fMRI from 30 participants, achieving an accuracy of 80.2 ± 4.7%, while the randomly initialized model failed to converge. We further validated the model’s performance on the Multiple Domain Task Dataset (MDTB), which contains fMRI data of 26 tasks from 24 participants. Thirteen tasks of fMRI were selected as inputs, and the results showed that the pre-trained model succeeded in classifying 11 of the 13 tasks. When using the 7 brain networks as input, variations of the performance were observed, with the visual network performed as well as whole brain inputs, while the limbic network almost failed in all 13 tasks.

**Discussion:**

Our results demonstrated the potential of self-supervised learning for fMRI analysis with small datasets and unpreprocessed data, and for analysis of the correlation between regional fMRI activity and cognitive tasks.

## 1. Introduction

Decoding brain activities is one of the most popular topics in neuroscience in recent years. One of the tasks of decoding brain activities is to predict the cognitive task states. In the past few years, deep learning has been used for brain decoding and achieved high accuracy (Huang et al., 2017; Li and Fan, 2019; Nguyen et al., 2020; Wang et al., 2020; Jiang et al., 2022). These deep models need large data amounts, but studies in fMRI are usually datasets with data from a few dozen individuals (Szucs and Ioannidis, 2020).

A method to solve this problem is transfer learning, which is to train a model on large datasets and transfer the parameters of the model to a small dataset (Koyamada et al., 2015; Zhang et al., 2018; Thomas et al., 2019). Self-supervised learning is a deep learning approach where models learn from unstructured or unlabeled data without explicit supervision (Mikolov et al., 2013), and has been used in various domains such as natural language processing and computer vision (Doersch and Zisserman, 2017). The core is to carefully design a self-supervised task that requires no manual annotation, yet pushes the model to extract useful characteristics from the data (Kumar et al., 2022). For instance, contrastive learning, a method in self-supervised learning, is a technique that enhances the performance of vision tasks by using the principle of contrasting samples against each other to learn attributes that are common between data classes. Like natural videos, fMRI data are high dimensional data containing both temporal and spatial information. The spatial and temporal autocorrelation is basic properties of fMRI signals and may explain numerous brain network topology features (Shinn et al., 2023). Unlike natural videos, the outlines of fMRI images rarely change, and the information is within the activity patterns of the voxels, i.e., the spatiotemporal variance of the values of voxels. Moreover, the fMRI data suffer from much lower signal to noise ratio (SNR) than natural video. Thus, researchers have employed various ways of self-supervised learning in fMRI: Li et al. (2023) used self-supervised learning method to calculate personalized brain functional networks fron fMRI data, and Thomas et al. (2022) modeled sequences of fMRI signals as sequences of text by natural language processing (NLP) self-supervised learning methods. Both studies employed self-supervised learning by internal persistency of brain networks, but it still remains an open question of whether we can build a simple end-to-end self-supervised model by the instance representations within 4-dimensional fMRI images.

Another challenge in applying deep learning to fMRI data is interpretability. Deep learning models are often criticized for their lack of interpretability, which can be problematic in fMRI studies where the underlying neurobiological mechanisms are of most interest. Researchers have used several methods to gain insight into how the network is processing the data: saliency maps (Simonyan et al., 2014), activation maximization (Erhan et al., 2009), and attentional mask (Jiang et al., 2022). All these are hard to make a direct link between the input data and performance. Inspired by multi-variate pattern analysis (MVPA) (Hanke et al., 2009), we figure out an input-dependent method for interpretability.

Inspired by these challenges, the main contributions to this paper are as follows. First, we propose a time-domain-based fMRI self-supervised learning method and validate it on small databases for downstream tasks. Second, we use fMRI signal in ROIs as input to explore the correlation between brain network activity and multi-domain cognitive tasks.

## 2. Materials and methods

### 2.1. Dataset

#### 2.1.1. HCP dataset

The minimally preprocessed 3 T data from the S1200 release of the Human Connectome Project (HCP) (Glasser et al., 2013) were used in this research. The pipeline of the minimally preprocessed removes spatial distortions, realigns volumes to compensate for subject motion, registers the fMRI data to the structural, reduces the bias field, normalizes the 4D image to a global mean, and masks the data with the final brain mask. The detail is described in Glasser et al. (2013). We employed the HCP task fMRI of 1,034 subjects during seven tasks: emotion, gambling, language, motor, relational, social, and working memory (WM). The HCP S1200 dataset has been minimally preprocessed with the HCP functional pipeline and normalized to the Montreal Neurological Institute’s (MNI) 152 space. According to the previous studies (Nguyen et al., 2020; Wang et al., 2020; Jiang et al., 2022), only one condition was selected for each task (Table 1). Similar to a previous study (Jiang et al., 2022), a bounding box of the size of [80, 96, 88] voxels was applied to each fMRI volume to crop out the blank parts within the images.

**Table 1:** Details of the selected HCP tasks and time series.

Task	Selected condition	Samples per subject	Frames of the block (time scale)
Emotion	Fear	2	26(18.72 s)
Gambling	Loss	2	39(28.08 s)
Language	Present story	2	29(20.88 s)
Motor	Right hand	2	17(12.24 s)
Relational	Relation	2	23(16.56 s)
Social	Mental	2	32(23.04 s)
Working Memory	2-back places	2	39(28.08 s)

#### 2.1.2. OpenNeuro dataset ds002938

To validate the self-supervised strategy in fMRI datasets of normal sample size, we employed the OpenNeuro dataset ds002938(Aben et al., 2022), which consists of fMRI data from 30 participants. The data were obtained in a 3 T Siemens MRI scanner, with 3.5 × 3.5 × 3.5 mm in-plane resolution and repetition time (TR) equal to 2 s. The details of the dataset and task are described in Aben et al. (2020). We employed the 1-back task scan, in which participants conducted a 1-back working memory task viewing grayscale images of 18 faces and 18 houses. The task is block-designed, with each block lasting for 36 s (18 TRs). Four-dimensional fMRI clips were segmented by covering the whole block and six extra TRs extended forward and backward (Table 2), and 8 clips were obtained for each condition.

**Table 2:** Details of the selected OpenNeuro ds002938 task and time series.

Task	Condition	Samples per subject	Frames of the block (time scale)
Working memory	Face	8	24(48 s)
Working memory	House	8	24(48 s)

#### 2.1.3. Multiple domain task battery dataset

Multiple domain task battery (MDTB) dataset (King et al., 2019) was used for validating the self-supervised strategy’s performance in fMRI data with multiple cognitive tasks of normal participant size. MDTB dataset contains fMRI data from 24 healthy individuals, conducting 26 tasks comprising 47 unique task conditions to engage a broad range of sensorimotor, cognitive and social/affective processes. The tasks were block-designed and measured over four fMRI scanning sessions (TR = 1 s). We excluded some non-block-design tasks, movie-watching tasks, rest task and short block tasks. We also selected nBackPic instead of verbal working memory for the nBack task, and we selected the motor sequence task and dropped the motor imagery task. Finally, thirteen tasks were selected, with one condition for each task (Table 3).

**Table 3:** Details of the selected MDTB task and time series.

Task	Selected condition	Task description	Samples per subject	Frames of the block (time scale)
Theory of Mind (ToM) (Dodell-Feder et al., 2011)	–	2AFC to indicate if short story contains true or false belief	16	35(35 s)
Action Observation (Observe) (Cross et al., 2012)	Video actions	Passive viewing of videos of knots being tied, learning the name of the knot (presented at top of screen) for a latter recall test.	16	20(20s)
ArithMetic (Arith) (Rickard et al., 2000)	Math	2AFC to indicate if simple multiplication equations are correct or incorrect	16	20(20s)
Object Viewing (ObjView)	–	Passive viewing, pictures of objects and a checkerboard pattern	16	35(35 s)
Biological Motion (BioMotion) (Troje, 2002)	Biological motion	2AFC to identify intact point-light walkers (either happy or sad)	16	20(20s)
Interval Timing (Interval) (Schubotz and von Cramon, 2001)	–	2AFC, indicating if a tone is short (100 ms) or long (175 ms)	16	30(30s)
Motor Sequence(Motor) (Wiestler and Diedrichsen, 2013)	Finger Sequence	6-element sequence, either requiring one key press with each of six fingers	16	19(19 s)
Object N-Back (NBack) (Owen et al., 2005)	2-back	2AFC, indicating if current stimulus in stream of objects matches objects displayed two items previously	16	35(35 s)
Response Alternatives (RespAlt) (Bischoff-Grethe et al., 2002)	Easy	Execute a fast motor response to an imperative signal (white cross) that appears 1 primed position	16	15(15 s)
Spatial Map (SpaMap)	Easy	Memorize a spatial mapping of 1 for subsequent recall	16	15(15 s)
Spatial Imagery (SpaImage) (Boly et al., 2007)	Spatial imagery	Imagine walking from room to room in childhood home, with a cue specifying the path to be taken	16	35(35 s)
Verb Generation (Verb) (Fiez, 1996)	Word reading	covert responses to visually presented nouns, repeating the stimulus	16	21(21 s)
Visual Search (Visual) (Donner et al., 2002)	Small	2AFC, indicating if target stimulus (“L”) is present among distractors (“T”), with set size = 4	16	15(15 s)

**Figure 1:**
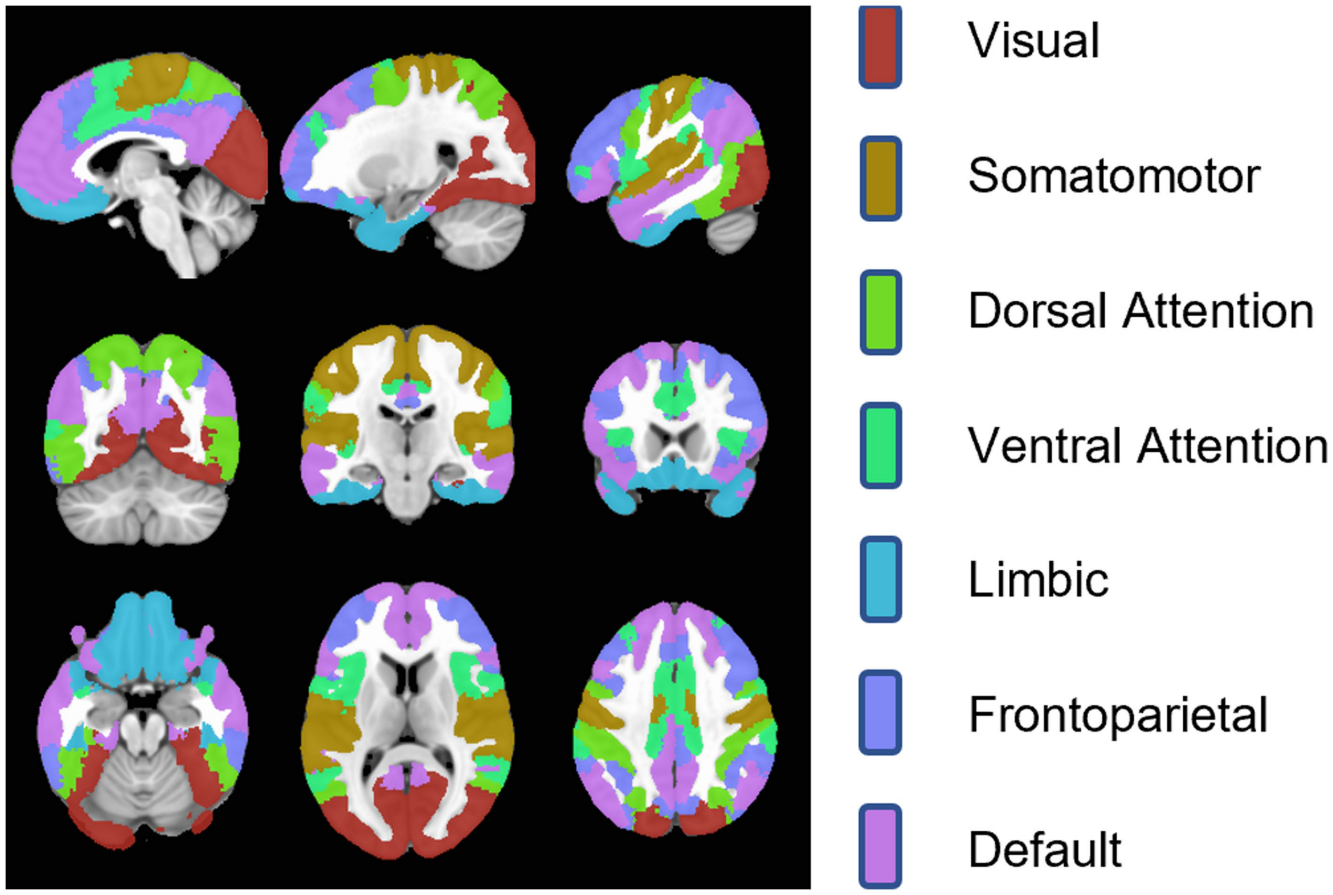
Seven brain networks in MNI152 space.

ROI-based analysis is a common method in MVPA (Hanke et al., 2009), and we also validate the ROI-based analysis of the deep model. The fMRI data were registered to MNI 152 spaces by the FMRIB Software Library (FSL) (Jenkinson et al., 2012), and then ROIs of 7 brain networks by Yeo et al. (2011) were used to obtain fMRI signal within each brain network (Figure 1). We trimmed the blank areas surrounding the segmented brain networks to save computational resources and expedite the processing speed (Table 3). Finally, four-dimensional fMRI clips were segmented by covering each block and 4 extra TRs extended forward and backward (Table 4).

**Table 4:** Bounding box size of 7 brain networks and the whole brain.

Brain network	Bounding box size
Visual	[60, 46, 47]
Somatomotor	[70, 36, 48]
Dorsal Attention	[66, 53, 53]
Ventral Attention	[68, 59, 50]
Limbic	[59, 54, 25]
Frontoparietal	[69, 59, 50]
Default	[78, 70, 57]
Whole Brain	[80, 96, 88]

### 2.2. The proposed network

**Figure 2:**
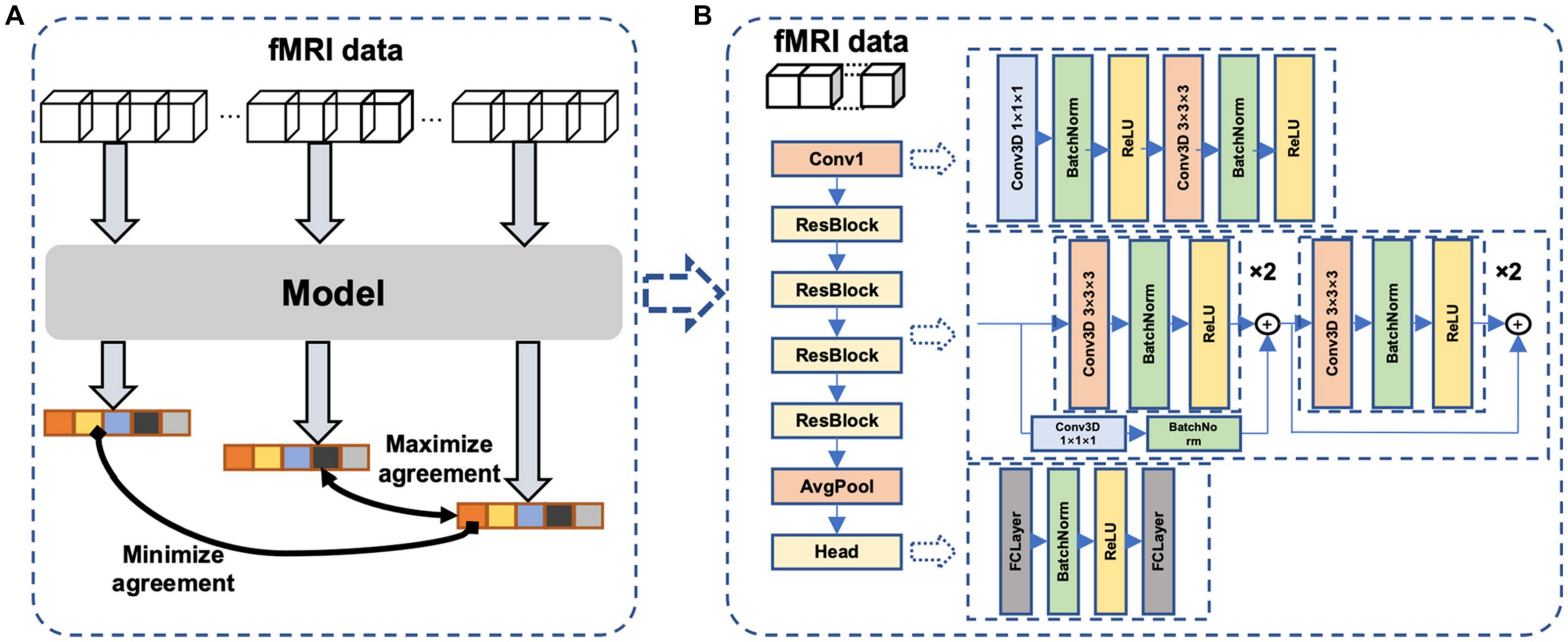
The proposed neural network. **(A)**The proposed framework of self-supervised learning. Dividing an fMRI signal segment into three parts: the beginning, middle, and end. After feature extraction and nonlinear mapping, the beginning and end parts are more widely separated, whereas the middle and end parts are closer in the contrast space. **(B)** the model consists of a temporal convolutional layer, four 3D residual layers, and a project head to map the feature to the contrast space.

The main idea of the proposed self-supervised method is the continuity of the human internal neural state, i.e., fMRI sequences that are temporally close exhibit a stronger correlation compared to sequences that are temporally distant. We thus design a temporal comparison loss within the contrast space (Figure 2A), and network parameters are updated accordingly to learn the potential temporal feature relationships in fMRI data. The proposed network (Figure 2B) comprises a temporal convolutional layer for incorporating temporal information, followed by four residual layers (He et al., 2016; Hara et al., 2018) to extract features and a non-linear layer for mapping these features to the contrast space.

#### 2.2.1. Loss function

Our core idea was inspired by contrastive learning, but with a novel approach: we utilized a single fMRI scan to generate positive and negative pairs, which differs from other contrastive learning methods. For a given fMRI signal, we segmented it into three sections: the beginning, middle, and end. We then utilized the end portion of the signal as the positive pair with the middle portion in the contrastive learning task, and the beginning portion with the end portion as the negative pair.

Based on this idea, we designed a loss function that is expressed as follows:


$$loss=-\log\left(\frac{e^{sim\left(y_{1},y_{3}\right)}}{e^{sim\left(y_{1},y_{3}\right)}+e^{sim\left(y_{2},y_{3}\right)}}\right)$$

Here, *y*_1_, *y*_2_, *y*_3_ is the output of the beginning, the middle and the end part of the input fMRI. The *sim*(*x*, *y*) function measures the cosine similarity of vectors *x* and *y*. The function *sim*(*x*, *y*) is defined as:


$$sim\left(x,y\right)=\frac{x*y}{{\left|\left|x\right|\right|}_{2}*{\left|\left|y\right|\right|}_{2}}$$

### 2.3. Training and evaluation

The model was constructed on PyTorch. The training was performed on an NVIDIA GTX 3090 graphic card. We choose 1,034 subjects from HCP dataset and 7 tasks were chosen for each subject. Two blocks were chosen for one task. That is to say, a total of 14,476 fMRI data were used. Among them, 5 tasks (Emotion, Gambling, Language, Social, Working Memory) were chosen to pretrain the model, so 10,340 fMRI data were used for pretraining. The left two tasks were used to execute a downstream classification task to validate the validity of the pretrained model. The reason we chose Motor and Relational tasks as downstream task is that these two task’s lengths are too short. The beginning and the end parts may overlap which we try to avoid. The batch size was set to 16 and each model was trained for 100 epochs using the Adam algorithm with the standard parameters (β1 = 0.9 and β2 = 0.999). The learning rate was initialized at 0.0001 and decayed by a factor of 0.99 for each training epoch. While pretraining the model, we pretrain the model with different input frames (frames = 9, 12, 15) as a comparison.

### 2.4. Downstream task

While conducting the downstream task, we reused the parameters of the model and added a fc layer as classifier in the end. A segment of k continuous frames, which was randomly split from each segment, was used as input for training. Meanwhile, the cross-entropy loss was used following the previous related works (Nguyen et al., 2020; Wang et al., 2020; Jiang et al., 2022). The cross-entropy loss is one of the mostly widely used loss function in classification for its convexity, probability estimation, heavy penalization of incorrect predictions, and multiple classes handling. Adam optimizer was used and the learning rate was set to 0.0001 and decayed by a factor of 10 when the validation accuracy plateaued after 10 epochs.

#### 2.4.1. Transfer learning within HCP (Motor and Relational tasks)

The pretrained model contains knowledge about the other 5 tasks, so we try to figure out if the model is helpful to the tasks whose type was not seen. Here, We also use a different number of subjects to finetune the model. Five-fold cross-subject validation was applied to minimize randomness in validation. The training epochs were set to 30 to avoid overfitting.

#### 2.4.2. Transfer learning to OpenNeuro ds002938

We fine-tuned a pre-trained model to a dataset consisting of working memory tasks of house and face images from 30 individuals. The scanning parameters, including TR and voxel size, were different from those of the HCP database, and the task types differed from the pre-training dataset for working memory tasks. Lastly, subject-based 5-fold cross-validation was performed. Due to transfer learning to a different dataset, the convergence of the pre-trained model is a bit slower compared to transferring the model to the HCP dataset. Thus, the fine-tuning epochs were set to 60 to get better convergence of the model.

#### 2.4.3. Transfer learning to multiple task dataset (cognitive function analysis based on brain networks)

Brain networks were used to decoding brain cognitive states. These networks are consistent with what we mentioned in section 2.1.3, including the visual network, somatomotor network, dorsal attention network, ventral attention network, limbic network, frontoparietal network, and default mode network. Most of the previous studies used to calculate the functional connections between different brain regions to calculate the features as the input of the neural network, while we directly input the entire brain region covered by the mask. Classification of cognitive function was performed on datasets consisting of 7 brain networks. Each of the network datasets was used as input for pre-training models for the classification task, and different results were obtained by utilizing various brain network inputs. Like transfer the model to the OpenNeuro ds002938, the fine-tuning epochs were set to 60.

## 3. Results

### 3.1. Transfer within the HCP dataset

**Figure 3:**
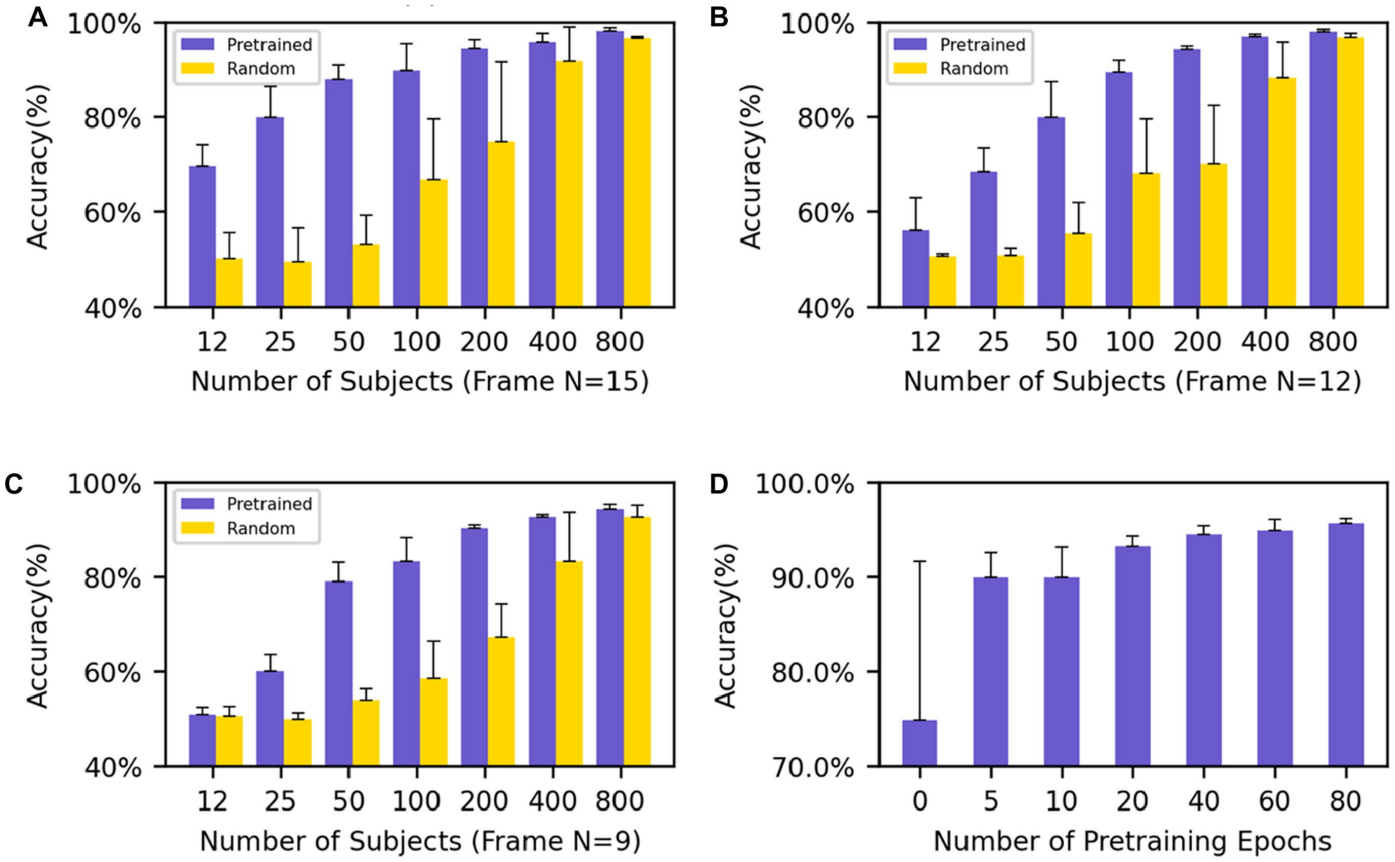
Performance evaluation on Motor and Relational tasks **(A–C)** show the average accuracy on the Motor and Relational classification task using different numbers of subjects to finetune the model and **(A–C)** use different frames as input (frame *N* = 15, 12, 9). **(D)** The average accuracy of different training epochs of the model which uses 200 subjects’ data to finetune. The performance goes better as the training process progresses.

The performance of various models was compared by the mean and standard deviation of accuracy. In the downstream classification tasks of Motor and Language, we investigated the impact of different input frame lengths and numbers of individuals on the results of fine-tuning the pre-trained models and compared them with randomly initialized models. The final classification results show that with an input frame length of 15 and model fine-tuning using 200 subjects, the model has an accuracy of 94.5 ± 1.8% while the randomly initialized model has an accuracy of 74.9 ± 16.7%, and the standard deviation of the pre-trained model is very low, showing the stable convergence of the model. The results (Figures 3A–C) showed that, under the same input frame length and a small dataset, the pre-trained model fine-tuning always outperformed the randomly initialized model. The models with an input length of 15 frames (Figure 3A) outperformed those with an input length of 12 and 9 frames (Figures 3B,C) with the same group of data. When the input frame length was 15, a good classification result (69.7 ± 4.4%) could be obtained even with only 12 individuals for fine-tuning. In contrast, even with 100 individuals for training, the model trained from scratch had difficulty in obtaining classification (66.8 ± 12.7%) higher than the random level (50%).

We also pretrained an Overlapped model with frame *N* = 9 and overlapped frame *N* = 3, where the middle part overlaped with the beginning and end parts by 3 frames while no overlaps existing between the beginning and end ones. The same downstream classification tasks (Motor-Relational) were applied and the results were shown in the Supplementary Table S1. The overplapped model performed comparable to the randomly initialized model, and worse than the non-overlapped model.

The results show that regardless of the amount of data used for fine-tuning, even if the network does not contain task-relevant information, good results can still be achieved compared to random initialization of the network. In particular, the network still shows good results and low standard deviation when fine-tuning with few subjects. At the same time, good results can still be obtained for networks with small input frame lengths. After only five epochs of pre-training, the model achieves an accuracy rate of 89.9 ± 2.6% with an input frame length of 15 and uses 200 subjects for the downstream task. As the training process deepens, the performance of the model when migrating to downstream tasks is increasingly better (Figure 3D).

### 3.2. Transfer to different datasets

The above experiment was conducted to validate the transferability of our self-supervised learning method using the same dataset as the downstream task. To further investigate whether our method can be applied across fMRI datasets with the site and scanning parameter differences, we conducted additional experiments using smaller datasets and a brain network-based dataset to explore the relationship between brain networks and cognition.

#### 3.2.1. Transfer to the OpenNeuro dataset ds002938

**Figure 4:**
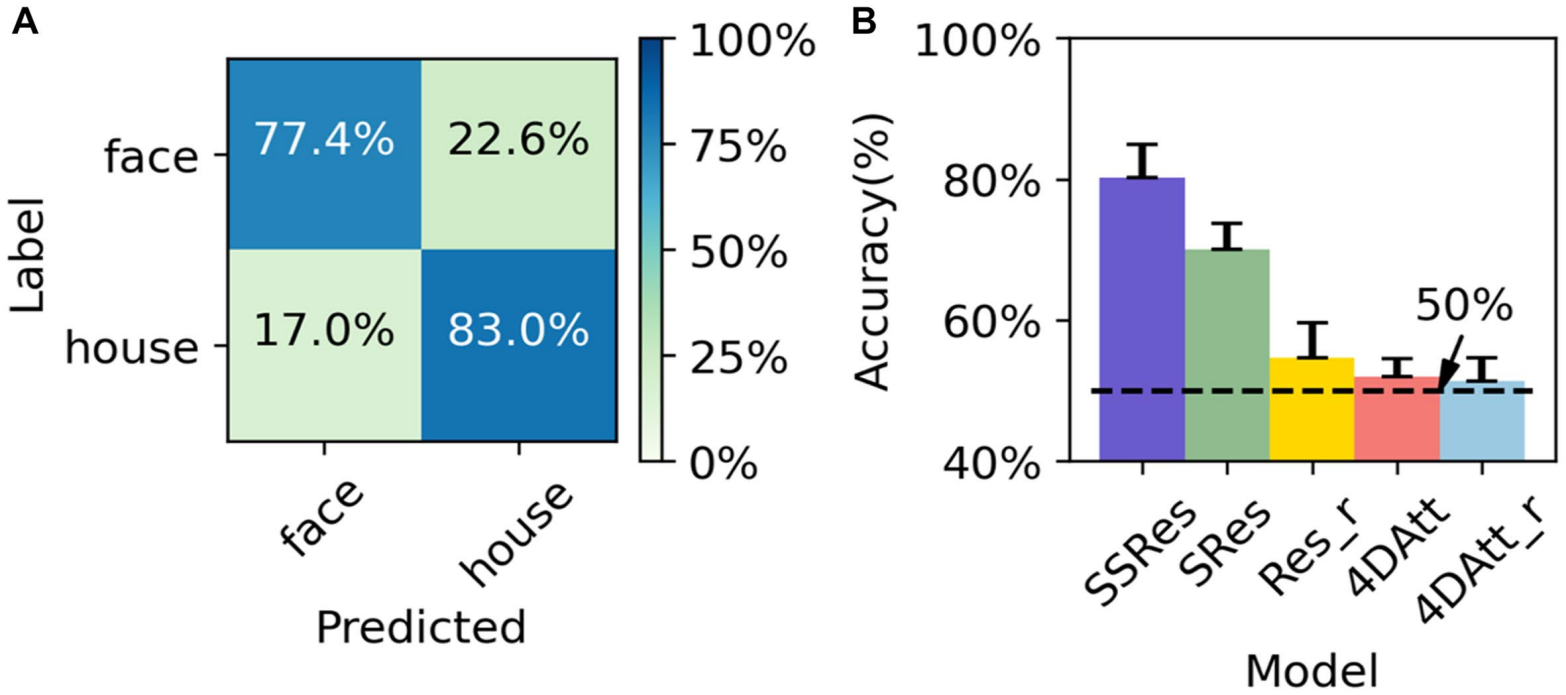
Performance evaluation on OpenNeuro ds002938 Dataset. **(A)** The average confusion matrix of OpenNeuro ds002938 task classification. **(B)** The performance of the different methods on OpenNeuro ds002938 dataset. SSRes means self-supervised resnet trained on the HCP dataset. SRes means supervised resnet trained on HCP. Res_r means resnet with randomly initialized. 4DAtt means 4D attention model trained on HCP. 4DAtt_r means 4D attention model with randomly initialized.

As a means of cross-site and scanning parameter validation, we utilized the OpenNeuro ds002938 dataset as an additional downstream task. The pre-trained model from HCP was applied to this dataset, and the results were compared with those obtained by randomly initialized parameters during training. Furthermore, the input data was employed in its unprocessed raw form and was not registered to either the standard MNI152 space or the subject’s own T1w. Like the previous experiment, we used the mean and standard deviation of accuracy as the criteria to measure the results and conducted five-fold cross-validation based on subjects to obtain the final results. The task is to perform sub-task between tasks. The transferred model achieved 80.2 ± 4.7% accuracy (Figure 4B) while the initial model trained from scratch failed to converge to a satisfactory accuracy (<55%) across a wide range of choices of hyper-parameter. And the model pretrained using supervised learning on HCP gets only 70 ± 3.4% correct in the classification task of this dataset, which is not as good as the generalization ability of the model trained by our proposed method.

#### 3.2.2. Transfer to the MDTB dataset

**Figure 5:**
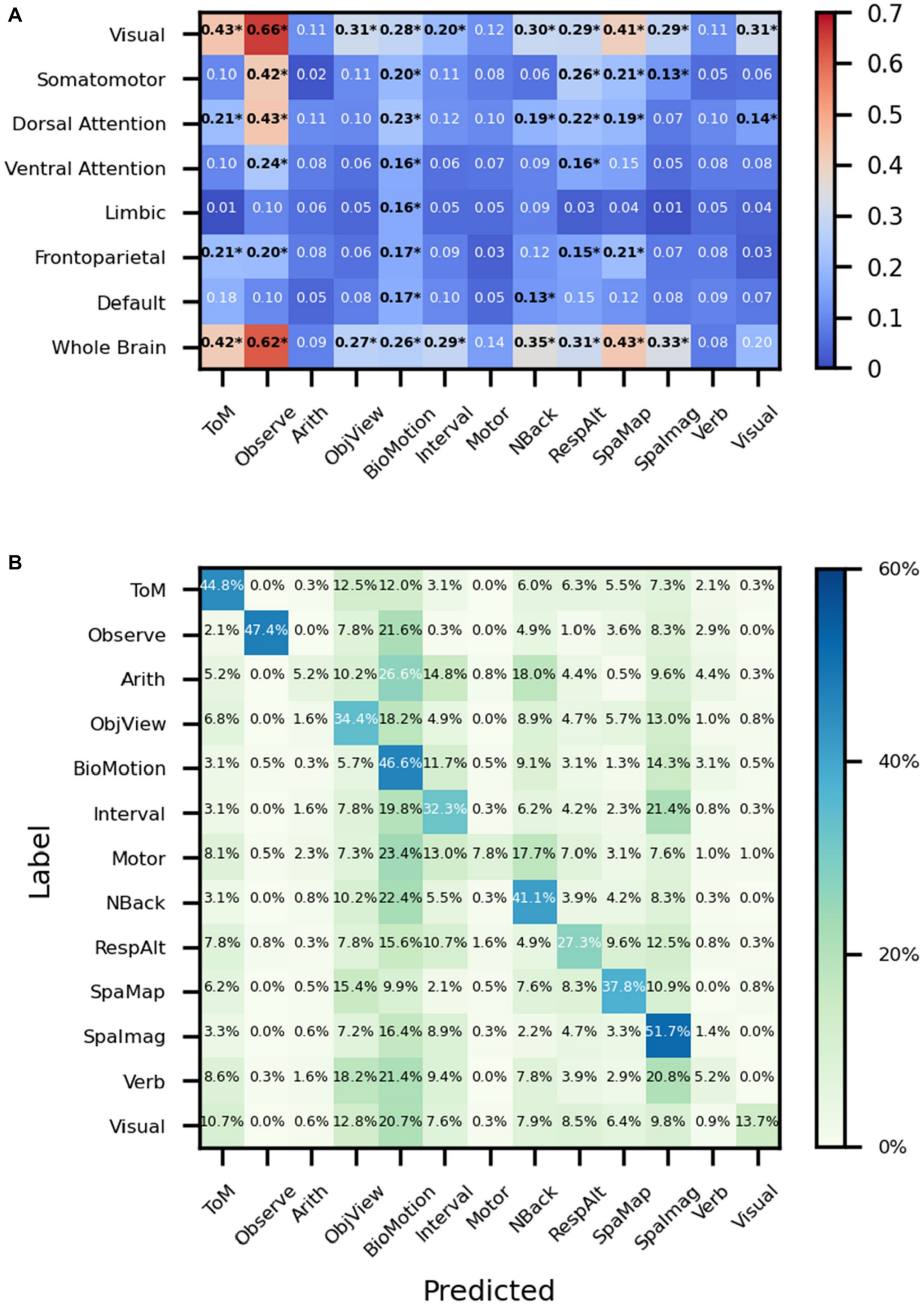
Performance on MDTB dataset. **(A)** The ‘*’ shows that after the *t*-test, the f1 scores were greater than the random classification level for *p* < 0.05 (with FDR corrected). ToM: Theory of Mind, Observe: Action Observation, Arith: ArithMetic, Object: Object Viewing, BioMotion: Biological Motion, Interval: Interval Timing, Object N-Back: NBack, Response Alternatives: ResAlt, Spatial Map: SpaMap, Spatial Imagery: SpaImag, Verb Generation: Verb, Visual Search: Visual. **(B)** The average confusion matrix of the task classification using the whole brain fMRI signal as input.

We try to use regions rather than volumes as input to explore whether the network works well even if only part of the brain’s information is preserved. F1 scores are calculated to measure the goodness of the results of the classification. For example, when the whole brain is used as input, the f1 scores of ToM, Observe, NBack, and SpaMap are 0.42, 0.62, 0.35, and 0.43, respectively, and when the visual network is used as input, the f1 scores of ToM, Observe, NBack, and SpaMap are 0.43, 0.66, 0.30, and 0.43, respectively. In particular, in the classification results, the f1 score was 0.31 when the visual network was used as input and the significance was verified by the false discovery rate (FDR) corrected t-test, while the f1 score did not pass the significance test when the whole brain signal was used as input The results show that the performance of task classification varies with the different brain networks used as input. Among them, the overall performance of the network was best when the visual network was used as input compared to other brain networks while the Limbic network was the input brain network, the results are basically at the random level except for the biological motion task. However, all brain regions, even the Somatomotor brain network, perform at random levels on the motor task (Figure 5A). What puzzled us was the poor classification results of the Motor task whether using whole-brain fMRI signals or fMRI signals from individual brain regions. We drew the average confusion matrix for the five-fold cross-validation of the whole-brain fMRI for the classification task (Figure 5B), and we found that the Motor task was misclassified as ToM, ObjetView, BioMot, Interval, NBack, RespAlt, and SpaImagery(Table 3).

## 4. Discussion

In this work, we proposed a self-supervised framework to learn internal spatiotemporal patterns within fMRI and allow the model to transfer to datasets of small size. When transferring the pre-trained model to the HCP dataset, the model shows higher accuracy and more robust results. In particular, when using the pre-trained model for the classification task on the OpenNeuro ds002938 dataset, we obtained an accuracy of 80.2 ± 4.7% using the raw data directly as input, while the randomly initialized model failed to converge. Finally, we use 7 brain networks as input to analyze the correlation between brain networks and cognitive tasks.

### 4.1. End-to-end network

The end-to-end training is robust, because the deep model may leverage information (like the signal patterns and individual brain structure) automatically. One challenge of end-to-end learning of fMRI is the big size of fMRI data, tens to hundreds of MB for one sample. Our work and related work (Wang et al., 2020; Jiang et al., 2022) have demonstrated that a not-too-deep neural network may be enough to abstract informative features in a normal game graphic card (such as NVidia GTX 1080Ti) with a batch size of around 10. Batch size matters in deep learning, and we will be happy if someone may answer whether a much bigger batch size improves the efficiency of deep learning, which we can not do at the present work due to the limited computing resources. We have proved that deep learning may learn in individual space, consistent with previous results (Jaiswal et al., 2021; Jiang et al., 2022). It means that the preprocessing of normalization to standard space may be removed from deep learning of fMRI, which is good for analysis that requires high efficiency (such as real-time fMRI). One might dream of a deep model robust to interferences, such as head motion, physiological noise, and signal drifts, and not requiring any preprocessing. Whether deep learning can achieve these remains an open question for futural works.

### 4.2. Transfer learning

Creating positive and negative pairs is a crucial step in contrastive learning, and it forms a fundamental component of the model construction. However, fMRI data exhibits distinct characteristics from natural images. The viewing angle of fMRI ‘videos’ is always the same, with a bounding box covering the whole brain. There are some self-supervised methods (Sermanet et al., 2018; Alwassel et al., 2020; Akbari et al., 2021) to extract features of videos, audio and text. While videos rely on visual elements to convey meaning, in fMRI data it is the spatiotemporal patterns of voxel value changes that carry significance. Previous work has proposed a self-supervised method that learns the dynamics of brain activity within brain networks by modeling sequences of activity akin to sequences of text (Thomas et al., 2022). The proposed contrastive pairs abandon spatial contrastive and focus on temporal information within the 4D fMRI data. The low SNR of fMRI means that the dominant signal within fMRI is the noise, not the internal neural signal (Bandettini, 2020). In the present work, positive and negative pairs are generated from clips of different temporal distances within the fMRI data, forcing the model to focus on long-term internal brain state variations but not short-term noises. This design also avoids the requirements for large batch sizes in contrastive learning and speeds up the training process.

Our results demonstrate that the HCP pre-trained model outperforms randomly initialized training for both Motor and Relational classification tasks, regardless of the amount of data used, and exhibits a shorter convergence time. Specifically, with only 12 subjects, the fine-tuned pre-trained model achieved impressive classification performance (69.7 ± 4.4%), indicating that the pre-training method allowed the network to learn general features of fMRI data. What is interesting is that a pre-trained model from HCP may help the learning of a small dataset (OpenNeuro dataset ds002938) of different sequences (multiband vs. single band), time of repetition (0.72 s vs. 2 s), spatial resolution (2 mm vs. 3.5 mm), and space (MNI152 vs. individual). The HCP pre-trained model learn from a dataset containing unpreprocessed fMRI from 30 participants, achieving an accuracy of 80.2 ± 4.7%, while the randomly initialized model failed to converge and even the supervised model trained on the HCP dataset only achieved an accuracy of 70 ± 3.7%. This suggests that self-supervised learning methods learn the spatiotemporal information inherent in fMRI data, ignoring the variance of scan setups, while supervised learning methods focus more on the task itself.

### 4.3. Interpretability

While using the MDTB dataset as input, we try to find the relationship between different brain networks and tasks. For the Biological Motion task, almost all networks show some decoding abilities (Figure 5A). Although the proposed model is biased to the predicted label of Biological Motion (Figure 5B), the f1 score used in the present study is not particularly sensitive to false positives or false negatives, as it takes both into account. The Biological Motion task in the MDTB is to identify whether intact point-light walkers are either happy or sad, which may engage emotion-related neural activity in a wider range of brain networks than common biological motion tasks. For the visual search task, the visual network showed the best results among all brain networks which is consistent with common sense perception. The proposed model misclassifies the motor sequence task into ToM, ObjView, BioMotion, Interval, NBack, RespAlt, and SpaImag (Figure 5B), most of which include key pressing in the task (Table 1) and lead to confusion with the finger pressing activity of the Motor task. And the f1 score of the visual search task was 0.31 when the visual network was used as input and the significance was verified by the FDR corrected t-test, while the f1 score did not pass the significance test when the whole brain signal was used as input. This suggests that using specific regional fMRI enables the neural network to focus on more detailed information.

Moreover, when the visual network is used as input, it performs the best among all brain networks, consistent with the visualization result of Wang et al. (2020), whose deep visualization highlights visual cortices for the 7 tasks in HCP. It indicates that for the decoding process of cognitive function tasks, the involvement of the visual network is necessary, and it may also be that different cognitive tasks have different activation patterns formed in the visual network by top-down feedback to the visual network from higher cognitive brain regions. The exact mechanism remains unclear and requires futural research. In conclusion, we used a brain network database to investigate the relationship between brain networks and cognitive functions. Our findings demonstrate that the pre-trained model is capable of extracting features not only from the entire brain volume as input but also ROIs as input, exhibiting its feature extraction and generalization abilities.

### 4.4. Limitations and future applications

In this paper, we propose an end-to-end fMRI temporal pre-training method that has shown good performance on downstream tasks. However, our approach also has some limitations that should be addressed. For instance, our method requires a relatively long block experiment design, and we solely relied on the HCP dataset as the pre-training dataset. While our downstream tasks demonstrate that our method can work well with different temporal resolutions, there is still a need for further exploration of how to combine datasets with varying temporal and spatial resolutions as the original pre-training dataset. Apply the model to resting-state and event-designed fMRI may also be possible. Furthermore, our use of a limited range of task types underscores the importance of a decoding model with a fine cognitive granularity that can generalize across multiple cognitive domains and brain states induced by various tasks. Meanwhile, the transformer (Vaswani et al., 2017) has shown competitive performance on several image and video classification tasks, and it deserves a try in fMRI data classification. Additionally, with the development of high-field super-resolution fMRI, high-resolution task-state fMRI-based data for brain decoding is expected in future studies.

## 5. Conclusion

This study proposes an end-to-end fMRI pretraining method that is based on fMRI internal temporal information. Through various downstream tasks, we demonstrate the effectiveness of our proposed method. Notably, our research on the OpenNeuro ds002938 dataset shows that the pre-trained model can learn from fMRI data of common sample size without preprocessing, thus simplifying the data analysis process. Moreover, we explore the relationship between brain networks and cognitive functions using different brain networks, highlighting the potential of deep learning in cognitive function brain region localization.

## Data availability statement

The original contributions presented in the study are included in the article/Supplementary material, further inquiries can be directed to the corresponding authors.

## Ethics statement

Ethical review and approval was not required for the study on human participants in accordance with the local legislation and institutional requirements. The patients/participants provided their written informed consent to participate in this study.

## Author contributions

CS, YaW, XW, and BQ ensured supervision over selecting the topic and research design. CS conducted the experiments and write the paper. RH and SC help process the fMRI data. MZ and YuW were in charge of process the results. All authors contributed to the article and approved the submitted version.

## Funding

This work was partially supported by National Natural Science Foundation of China (21876041) and the University Synergy Innovation Program of Anhui Province (GXXT-2021-003).

## Conflict of interest

The authors declare that the research was conducted in the absence of any commercial or financial relationships that could be construed as a potential conflict of interest.

## Publisher’s note

All claims expressed in this article are solely those of the authors and do not necessarily represent those of their affiliated organizations, or those of the publisher, the editors and the reviewers. Any product that may be evaluated in this article, or claim that may be made by its manufacturer, is not guaranteed or endorsed by the publisher.

## Supplementary material

The Supplementary material for this article can be found online at: https://www.frontiersin.org/articles/10.3389/fnins.2023.1199312/full#supplementary-material

## References

[ref1] Aben B. Calderon C. B. Bussche E. V. Verguts T. (2022). Task-dependent effort-induced connectivity. OpenNeuro.

[ref2] Aben B. Calderon C. B. Van den Bussche E. Verguts T. (2020). Cognitive effort modulates connectivity between dorsal anterior cingulate cortex and task-relevant cortical areas. J. Neurosci. 40, 3838–3848. doi: 10.1523/JNEUROSCI.2948-19.2020, PMID: 32273486 PMC7204076

[ref3] Akbari H. Yuan L. Qian R. Chuang W.-H. Chang S.-F. Cui Y.. (2021). Vatt: transformers for multimodal self-supervised learning from raw video, audio and text. Adv. Neural Inf. Proces. Syst. 34, 24206–24221.

[ref4] Alwassel H. Mahajan D. Korbar B. Torresani L. Ghanem B. Tran D. (2020). Self-supervised learning by cross-modal audio-video clustering. Adv. Neural Inf. Proces. Syst. 33, 9758–9770.

[ref5] Bandettini P. A. (2020). Fmri Cambridge, MA: MIT Press

[ref6] Bischoff-Grethe A. Ivry R. B. Grafton S. T. (2002). Cerebellar involvement in response reassignment rather than attention. J. Neurosci. 22, 546–553. doi: 10.1523/JNEUROSCI.22-02-00546.2002, PMID: 11784801 PMC6758676

[ref7] Boly M. Coleman M. R. Davis M. H. Hampshire A. Bor D. Moonen G.. (2007). When thoughts become action: an fMRI paradigm to study volitional brain activity in non-communicative brain injured patients. NeuroImage 36, 979–992. doi: 10.1016/j.neuroimage.2007.02.047, PMID: 17509898

[ref8] Cross E. S. Cohen N. R. Hamilton A. F. D. Ramsey R. Wolford G. Grafton S. T. (2012). Physical experience leads to enhanced object perception in parietal cortex: insights from knot tying. Neuropsychologia 50, 3207–3217. doi: 10.1016/j.neuropsychologia.2012.09.028, PMID: 23022108 PMC3588172

[ref9] Dodell-Feder D. Koster-Hale J. Bedny M. Saxe R. (2011). fMRI item analysis in a theory of mind task. NeuroImage 55, 705–712. doi: 10.1016/j.neuroimage.2010.12.040, PMID: 21182967

[ref10] Doersch C. Zisserman A. (2017). Multi-task self-supervised visual learning. Proceedings of the IEEE international conference on computer vision, Venice, Italy

[ref11] Donner T. H. Kettermann A. Diesch E. Ostendorf F. Villringer A. Brandt S. A. (2002). Visual feature and conjunction searches of equal difficulty engage only partially overlapping frontoparietal networks. NeuroImage 15, 16–25. doi: 10.1006/nimg.2001.0951, PMID: 11771970

[ref12] Erhan D. Bengio Y. Courville A. Vincent P. (2009). *Visualizing higher-layer features of a deep network*. Technical report, University of Montreal, Montreal.

[ref13] Fiez J. A. (1996). Cerebellar contributions to cognition. Neuron 16, 13–15. doi: 10.1016/S0896-6273(00)80018-5 8562076

[ref14] Glasser M. F. Sotiropoulos S. N. Wilson J. A. Coalson T. S. Fischl B. Andersson J. L.. (2013). The minimal preprocessing pipelines for the human connectome project. NeuroImage 80, 105–124. doi: 10.1016/j.neuroimage.2013.04.127 23668970 PMC3720813

[ref15] Hanke M. Halchenko Y. O. Sederberg P. B. Hanson S. J. Haxby J. V. Pollmann S. (2009). PyMVPA: a Python toolbox for multivariate pattern analysis of fMRI data. Neuroinformatics 7, 37–53. doi: 10.1007/s12021-008-9041-y, PMID: 19184561 PMC2664559

[ref16] Hara K. Kataoka H. Satoh Y. (2018). Can spatiotemporal 3d cnns retrace the history of 2d cnns and imagenet? Proceedings of the IEEE conference on computer vision and pattern recognition Piscataway: IEEE.

[ref17] He K. Zhang X. Ren S. Sun J. (2016). Deep residual learning for image recognition. Proceedings of the IEEE conference on computer vision and pattern recognition Piscataway: IEEE.

[ref18] Huang H. Hu X. Zhao Y. Makkie M. Dong Q. Zhao S.. (2017). Modeling task fMRI data via deep convolutional autoencoder. IEEE Trans. Med. Imaging 37, 1551–1561. doi: 10.1109/TMI.2017.2715285, PMID: 28641247

[ref19] Jaiswal A. Babu A. R. Zadeh M. Z. Makedon F. Wylie G. (2021). “Understanding cognitive fatigue from fMRI scans with self-supervised learning” in arXiv preprint arXiv:2106.15009

[ref20] Jenkinson M. Beckmann C. F. Behrens T. E. Woolrich M. W. Smith S. M. (2012). Fsl. NeuroImage 62, 782–790. doi: 10.1016/j.neuroimage.2011.09.015 21979382

[ref21] Jiang Z. F. Wang Y. M. Shi C. W. Wu Y. Y. Hu R. J. Chen S. S.. (2022). Attention module improves both performance and interpretability of four-dimensional functional magnetic resonance imaging decoding neural network. Hum. Brain Mapp. 43, 2683–2692. doi: 10.1002/hbm.25813, PMID: 35212436 PMC9057093

[ref22] King M. Hernandez-Castillo C. R. Poldrack R. A. Ivry R. B. Diedrichsen J. (2019). Functional boundaries in the human cerebellum revealed by a multi-domain task battery. Nat. Neurosci. 22, 1371–1378. doi: 10.1038/s41593-019-0436-x, PMID: 31285616 PMC8312478

[ref23] Koyamada S. Shikauchi Y. Nakae K. Koyama M. Ishii S. (2015). Deep learning of fMRI big data: a novel approach to subject-transfer decoding. arXiv [Preprint]. Available at: https://arxiv.org/abs/1502.00093

[ref24] Kumar P. Rawat P. Chauhan S. (2022). Contrastive self-supervised learning: review, progress, challenges and future research directions. Int. J. Multimed. Informat. Retr. 11, 461–488. doi: 10.1007/s13735-022-00245-6

[ref25] Li H. M. Fan Y. (2019). Interpretable, highly accurate brain decoding of subtly distinct brain states from functional MRI using intrinsic functional networks and long short-term memory recurrent neural networks. NeuroImage 202:116059. doi: 10.1016/j.neuroimage.2019.116059, PMID: 31362049 PMC6819260

[ref26] Li H. Srinivasan D. Zhuo C. Cui Z. Gur R. E. Gur R. C.. (2023). Computing personalized brain functional networks from fMRI using self-supervised deep learning. Med. Image Anal. 85:102756. doi: 10.1016/j.media.2023.102756 PMC10103143 36706636

[ref27] Mikolov T. Sutskever I. Chen K. Corrado G. S. Dean J. (2013). Distributed representations of words and phrases and their compositionality. Adv. Neural Inf. Proces. Syst. 26

[ref28] Nguyen S. Ng B. Kaplan A. D. Ray P. (2020). Attend and decode: 4d fmri task state decoding using attention models. Proc. Mach. Learn. Res. 136, 267–279.

[ref29] Owen A. M. McMillan K. M. Laird A. R. Bullmore E. T. (2005). N-back working memory paradigm: a meta-analysis of normative functional neuroimaging. Hum. Brain Mapp. 25, 46–59. doi: 10.1002/hbm.20131, PMID: 15846822 PMC6871745

[ref30] Rickard T. C. Romero S. G. Basso G. Wharton C. Flitman S. Grafman J. (2000). The calculating brain: an fMRI study. Neuropsychologia 38, 325–335. doi: 10.1016/S0028-3932(99)00068-8, PMID: 10678698

[ref31] Schubotz R. I. von Cramon D. Y. (2001). Interval and ordinal properties of sequences are associated with distinct premotor areas. Cereb. Cortex 11, 210–222. doi: 10.1093/cercor/11.3.210, PMID: 11230093

[ref32] Sermanet P. Lynch C. Chebotar Y. Hsu J. Jang E. Schaal S.. (2018). Time-contrastive networks: self-supervised learning from video. 2018 IEEE international conference on robotics and automation (ICRA) Piscataway: IEEE.

[ref33] Shinn M. Hu A. Turner L. Noble S. Preller K. H. Ji J. L.. (2023). Functional brain networks reflect spatial and temporal autocorrelation. Nat. Neurosci. 26, 867–878. doi: 10.1038/s41593-023-01299-3, PMID: 37095399

[ref34] Simonyan K. Vedaldi A. Zisserman A. (2014). Deep inside convolutional networks: visualising image classification models and saliency maps. Proceedings of the international conference on learning representations (ICLR), ICLR.

[ref35] Szucs D. Ioannidis J. P. A. (2020). Sample size evolution in neuroimaging research: an evaluation of highly-cited studies (1990–2012) and of latest practices (2017–2018) in high-impact journals. NeuroImage 221:117164. doi: 10.1016/j.neuroimage.2020.117164, PMID: 32679253

[ref36] Thomas A. W. Müller K.-R. Samek W. (2019). Deep transfer learning for whole-brain FMRI analyses. OR 2.0 context-aware operating theaters and machine learning in clinical neuroimaging: second international workshop, OR 2.0 2019, and second international workshop, MLCN 2019, held in conjunction with MICCAI 2019, Shenzhen, China, October 13 and 17, 2019, Proceedings 2, Springer.

[ref37] Thomas A. Ré C. Poldrack R. (2022). Self-supervised learning of brain dynamics from broad neuroimaging data. Adv. Neural Inf. Proces. Syst. 35, 21255–21269.

[ref38] Troje N. F. (2002). Decomposing biological motion: a framework for analysis and synthesis of human gait patterns. J. Vis. 2, 371–387. doi: 10.1167/2.5.2, PMID: 12678652

[ref39] Vaswani A. Shazeer N. Parmar N. Uszkoreit J. Jones L. Gomez A. N.. (2017). Attention is all you need. Adv. Neural Inf. Proces. Syst. 30

[ref40] Wang X. X. Liang X. Jiang Z. F. Nguchu B. A. Zhou Y. W. Wang Y. M.. (2020). Decoding and mapping task states of the human brain via deep learning. Hum. Brain Mapp. 41, 1505–1519. doi: 10.1002/hbm.24891, PMID: 31816152 PMC7267978

[ref41] Wiestler T. Diedrichsen J. (2013). Skill learning strengthens cortical representations of motor sequences. elife 2:e00801. doi: 10.7554/eLife.00801, PMID: 23853714 PMC3707182

[ref42] Yeo B. T. T. Krienen F. M. Sepulcre J. Sabuncu M. R. Lashkari D. Hollinshead M.. (2011). The organization of the human cerebral cortex estimated by intrinsic functional connectivity. J. Neurophysiol. 106, 1125–1165. doi: 10.1152/jn.00338.2011, PMID: 21653723 PMC3174820

[ref43] Zhang H. Chen P.-H. Ramadge P. (2018). Transfer learning on fMRI datasets. Paper presented at 21st International Conference on Artificial Intelligence and Statistics, AISTATS 2018, Playa Blanca, Lanzarote, Canary Islands, Spain

