# Supplementary material for: Self-supervised pretraining improves the performance of classification of task functional magnetic resonance imaging
Source: Front Neurosci. 2023 Jun 26;17:1199312. doi: 10.3389/fnins.2023.1199312 (PMC10330812; doi:10.3389/fnins.2023.1199312)
Supplement: Supplementary file 1 [file Data_Sheet_1.PDF]

Table S1 average accuracy of non-overlapped and overlapped setting

Number of Subjects	Non-overlapped	Overlapped	Random initialized
12	50.3±1.6%	51.1±2.6%	50.9±1.1%
25	60.0±4.9%	50.1±0.8%	50.1±1.3%
50	78.9±4.1%	51.3±1.2%	54.0±2.3%
100	83.2±5.0%	57.0±2.1%	58.4±7.9%
200	90.3±0.6%	64.3±4.4%	67.2±7.1%
400	92.5±0.4%	85.1±2.0%	83.1±10.5%
800	94.2±1.0%	91.8±0.7%	92.5±2.6%
